# Intrahepatic hematoma secondary to transjugular intrahepatic portosystemic stent–shunt procedure: Case report and literature review

**DOI:** 10.1097/MD.0000000000031753

**Published:** 2022-11-11

**Authors:** Ziyao Cheng, Jiayu Ju, Qingliang Zhu, Mingming Deng, Hailong Zhang

**Affiliations:** a Department of Gastroenterology, The Affiliated Hospital of Southwest Medical University, Luzhou, Sichuan Province, China.

**Keywords:** hepatic pseudoaneurysm, intrahepatic hematoma, TIPSS-related complications, transjugular intrahepatic portosystemic stent–shunt

## Abstract

Transjugular intrahepatic portosystemic stent–shunt (TIPSS) is a minimally invasive procedure used for the treatment of portal hypertension. Intrahepatic hematoma is the rare complication caused by hepatic arterial injuries from TIPSS procedure.

**Patient concerns:**

This case report illustrated a 77-year-old man with hepatitis B virus-induced cirrhosis who underwent TIPSS.

**Diagnoses:**

The patient suffered from intrahepatic hematoma and hepatic pseudoaneurysm because of the hepatic artery injury after TIPSS.

**Interventions:**

The hepatic artery laceration began at the level of the branch of the left hepatic artery was embolized.

**Outcomes:**

The acute intrahepatic hematoma and hepatic pseudoaneurysm of the patient were cured.

**Conclusion:**

In this report, we describe a cirrhosis patient with a large intrahepatic hematoma secondary to TIPSS, and a literature review is also presented. The intrahepatic hematoma and hepatic pseudoaneurysm should be paid more attention after TIPSS while early-stage prevention should be carried out.

## 1. Introduction

Transjugular intrahepatic portosystemic stent–shunt (TIPSS) has been in use to treat complications of portal hypertension for more than 30 years.^[1]^ A rare but life-threating complication of this procedure is intrahepatic hematoma caused by hepatic arterial injuries. Treatment options for this condition generally include conservative therapy, transcatheter arterial embolization, and surgery. Thus far, there are no literature review on this rare complication. Herein, we present a cirrhosis patient with a large intrahepatic hematoma secondary to TIPSS procedure treated with transcatheter arterial embolization, and a literature review is also presented.

## 2. Case presentation

A 77-year-old man with hepatitis B virus-induced cirrhosis was referred to our unit, who presented with melena and shortness of breath. Upon physical examination, his cardiovascular apparatuses were without alterations, the abdomen was flaccid, with a liver 7 cm from the right costal edge. The peritoneocentesis and thoracocentesis examination both revealed that no tumor cell was found. The pleural fluid was sterile and transudate with a serum-fluid albumin gradient > 1.1 g/dL, consistent with hydrothorax. Routine laboratory examination showed hemoglobin 82 g/L, platelets 95 × 10^9^/L, total bilirubin 10.8 umol/L, albumin 26.9 g/L, prothrombin time 12.9s, alanine transaminase (ALT) 21 IU/L, and aspartate transaminase (AST) 46IU/L. A triple-phase computed tomography (CT) scan demonstrated signs of portal hypertension, moderate pleural effusion on right side with a small quantity on the other side. Ascites was managed with diuretics and salt restriction. Drainage of pleural cavity was tried; however, this was unsuccessful.

Due to hepatic hydrothorax and secondary prevention for variceal bleeding, a TIPSS implant was agreed on and performed. During TIPSS procedure, TIPSS needle assembly (Rupus-100, Cook Incorporated, Bloomington) was advanced into the liver parenchyma under fluoroscopic guidance. A total of 3 passes of the inner needle were made before a central portal vein branch was punctured. No sign of puncture into hepatic arteries, bile ducts, or extrahepatic puncture was seen. A 10 mm × 8 cm bare stent (Cordis, Florida) was placed between the middle hepatic vein and the left branch of the portal vein. By expanding the stent through an 8 mm × 6 cm balloon catheter (Cordis Europa N.V, Roden, Netherlands), the portosystemic pressure gradient between the portal vein and inferior vena cava decreased from 24 to 11 mmHg. The patient presently received a bolus of 2000 U heparin intravenously for prophylaxis of stent thrombosis and was then started on low molecular weight heparin at a 0.4-mL q12h for 3 days.

**Figure 1.**
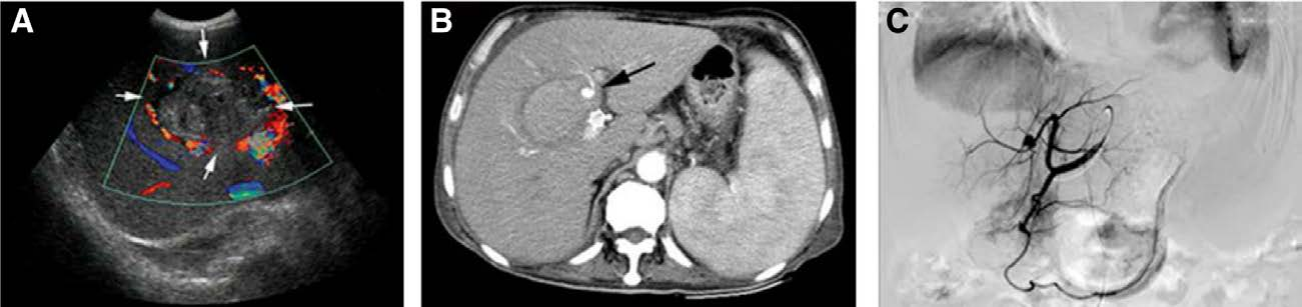
Images of the liver 3 days after TIPSS insertion. (A) Abdominal Doppler Ultrasonography showed a 8.6 × 5.4 cm hybrid echogenic mass in porta hepatis with no blood flow (white arrow). (B) Abdominal CT scan showed the presence of a 5.5 × 5.34 cm intrahepatic hematoma and a left hepatic pseudoaneurysm (black arrow). (C) Celiac angiography performed 48 h after onset of the hematoma and confirmed the hepatic pseudoaneurysm. The hepatic artery laceration began at the level of the branch of the left hepatic artery. The right hepatic artery which originated from superior mesenteric artery was normal.

The third evening after the procedure, he complained of severe upper abdominal pain with mild liver tenderness. The vital signs were stable, and no signs of peritoneal irritation were evident. Routine examination gave the following results: hemoglobin 76 g/L, platelets 86 × 10^9^/L, prothrombin time 13.1 s, activated partial thromboplastin time 46.8 s, total bilirubin 16.6 umol/L, albumin 23.6 g/L, ALT 36 IU/L, and AST 61 IU/L. Abdominal Doppler Ultrasonography showed a 8.6 × 5.4 cm hybrid echogenic mass in porta hepatis with no blood flow (Fig. 1). An abdominal CT scan was performed and showed the presence of a 5.5 × 5.34 cm intrahepatic hematoma and a left hepatic pseudoaneurysm.

**Figure 2.**
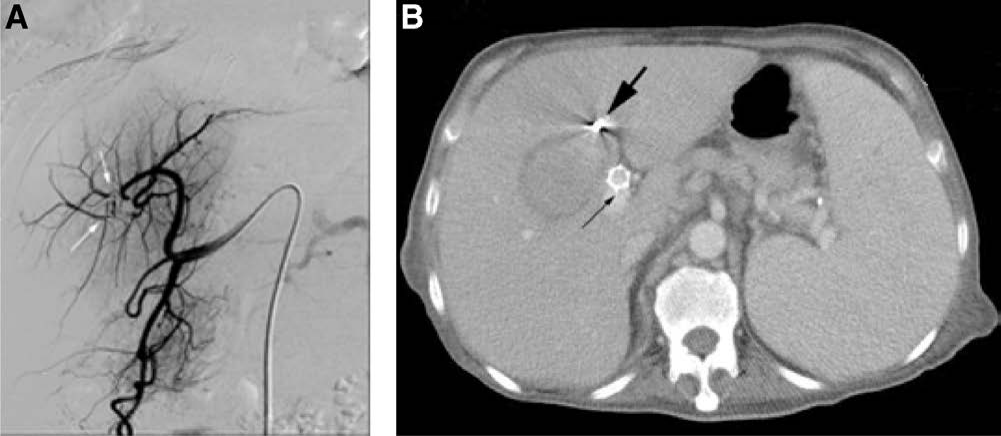
Images after embolization of the pseudoaneurysm with micro coils. (A) Hepatic angiography confirmed embolization of the pseudoaneurysm with micro coils (white arrows). (B) CT scan indicated occlusion of the pseudoaneurysm (black arrow).

Celiac angiography was performed 48 h after onset of the hematoma and confirmed a hepatic pseudoaneurysm. The hepatic artery laceration began at the level of the branch of the left hepatic artery. The right hepatic artery which originated from superior mesenteric artery was normal. Micro catheter (Cook, Bloomington, IN) was advanced into the left hepatic artery. The precise margins of the hepatic artery laceration were identified by microcatheter angiography on left anterior oblique. Micro coils (Cook, Bloomington, IN) were inserted for embolizing the laceration. Final angiography showed no filling of the pseudoaneurysm (Fig. 2).

The clinical course was favorable. The patient’s urine increased from 1200 mL/d up to 2800 mL/d. Two days after embolization management, the hepatic biological tests showed total bilirubin 20.1 umol/L, ALT 29 IU/L, AST 66 IU/L, as the same level of pre-TIPSS level. An abdominal CT scan realized coil emboli to the pseudoaneurysm and gradual resolution of the hematoma.

## 3. Discussion

Major complications caused by hepatic arterial injuries from TIPSS procedures are generally rare, including intrahepatic hematoma, hepatic arterial pseudoaneurysm, arterio-portal fistulae, arterio-venous fistulae, hemobilia, and intraabdominal bleeding.^[2–5]^ Schweiger et al^[6]^ first reported an intrahepatic hematoma secondary to TIPSS in a Budd-Chiari syndrome (BCS) patient. Since then, only 16 cases have been described in English literatures (Table 1). Although the prognosis of intrahepatic hematoma is good, but death to rupture of a hematoma has been reported.^[12]^ So, it is essential for us to note this rare complication.

**Table 1:** Summary of reported cases of intrahepatic hematoma due to transjugular intrahepatic portosystemic stent–shunt procedure.

Author/Reference/Year	Etiology	Number of cases	Imaging (Ultrasound/CT/MRI)
Gazzera^[7]^ (2012)	Cirrhosis	3	Intrahepatic hematoma
Han^[8]^ (2011)	Cirrhosis	1	Intrahepatic hematoma
Terreni^[9]^ (2007)	BCS	1	Intrahepatic hematoma^a^
Tripathi^[10]^ (2006)	Not available	1	Intrahepatic hematoma
Rössle^[11]^ (2004)	BCS	2	Intrahepatic hematoma
Mancuso^[12]^ (2003)	BCS	2	Intrahepatic hematoma
Fickert^[13]^ (2000)	BCS	1	Intrahepatic hematoma^a^
Williams^[14]^ (1998)	Cirrhosis	1	Intrahepatic hematoma
Urata^[15]^ (1998)	Cirrhosis	1	Intrahepatic hematoma
Merli^[16]^ (1998)	Cirrhosis	1	Intrahepatic hematoma
Hasegawa^[17]^ (1998)	BCS	1	Intrahepatic hematoma and active bleeding^a^
Schweiger^[6]^ (1997)	BCS	1	Intrahepatic hematoma and pseudoaneurysm^a^

BCS = Budd-Chiari syndrome, CT = computed tomography, MRI = magnetic resonance imaging.

a Selective angiography was performed.

The incidence of intrahepatic hematoma from TIPSS procedures is not exactly clear. In a large series of 658 patients who received TIPSS for complications of portal hypertension, intrahepatic hematoma was observed in 2 cases.^[18]^ In another series consisting of 67 portal hypertension patients treated with TIPSS, the incidence of intrahepatic hematoma was 1.5%.^[12]^ The difference may be caused by the asymptomatic nature of most intrahepatic hematoma and their tendency to resolve spontaneously. However, this incidence may be underestimated, because post-TIPSS scan are not routinely performed.

There are several factors affecting intrahepatic hematoma occurring due to TIPSS procedure. An important precipitating factor is routinely long-term anticoagulation in BCS patients. BCS patients may meet higher occlusion after TIPSS due to hypercoagulable states. It is now generally accepted that patients with BCS may benefit from long-term anticoagulation treatment.^[19]^ However, in all series that reported intrahepatic hematoma, the incidence in BCS patients was much higher than that in cirrhosis patients.^[3,20]^ Review of the literatures revealed that 8 of 16 patients with hematoma presented were diagnosed as BCS, and 7 received long-term anticoagulation after TIPSS. Because the number of patients received TIPSS with cirrhosis is far more than those with BCS, the incidence of intrahepatic hematoma is much higher in BCS patients. Gazzera et al^[18]^ also reported 2 cases of hematoma after TIPSS procedures, both in heavily anticoagulated patients with cirrhosis. Therefore, it has been suggested that anticoagulation is a potential risk of hematoma occurring. In addition, increased trauma to liver during technically challenging procedures and existing hypertension may also affect hematoma occurring.^[21,22]^ Whether or not the diameter of TIPSS needle affect hematoma formation has not been investigated. In our case, although no sign of hepatic artery injuries were seen during TIPSS procedure, many attempts causing potential trauma to liver before puncturing into portal vein and anticoagulation after TIPSS may be the precipitating factors for hematoma occurring.

Generally, hepatic arterial injuries usually spontaneously resolves and do not cause severe consequences.^[5,18]^ The symptom of intrahepatic hematoma not only depends on its size, but also perhaps upon the pain threshold of the patient. Clinically symptomatic intrahepatic hematoma during or after TIPSS procedure may present sudden right upper abdominal pain, which is frequently the only clue to suspect hematoma occurring (Table 2).^[23]^ The massive blood accumulation in liver cause compression-induced ischemic injury, resulting in dramatic increase in liver enzymes, decrease in hemoglobin. Death due to hematoma rupture was also reported. The time of hematoma occurring after TIPSS procedure is not well defined. Three cases with BCS developed a large hematoma on days 10, 11, 13 following TIPSS,^[9,13,17]^ our case presented hematoma on day 3. The shortest and longest time of hematoma occurring after TIPSS is not well-known.

**Table 2:** Details of patients with intrahepatic hematoma.

Etiology	Number of patients	Anticoagulation	Symptom	Treatment	Prognosis
BCS	8	7 received	5^a^ with sudden right abdominal pain	4 with TAE therapy, the pseudoaneurysm was embolized	All were good, except 1 died for liver rupture
			3 Not available	4 Not available	
Cirrhosis	7	2^b^ received	Not available	Not available	All were good

TAE = transcatheter arterial embolization.

a The symptom was reported in references.^[6,9,12,13,17]^

b Whether or not the other 5 patients received anticoagulation were not available.

When hematoma occurs, pseudoaneurysm and active bleeding should be actively sought, because treatment may be absolutely different when they exist. Ultrasound and enhanced CT or magnetic resonance imaging (MRI) have great value in patients suspected with hematoma,^[17]^ as demonstrated by Table 1. Selective angiography is the golden criteria of hepatic arterial injuries and should be done if pseudoaneurysm or active bleeding is suspected by ultrasound and enhanced CT or MRI. So far, only one BCS patient with pseudoaneurysm formation has been reported in English literature.^[6]^ Our case is the first report of pseudoaneurysm formation in cirrhosis patients secondary to TIPSS.

Patients with intrahepatic hematoma usually have favorable prognosis after treatment. However, as intrahepatic hematoma often causes hemorrhagic shock and is vulnerable to infections, conservative management, including analgesics, antibiotics, fluid replacement, transfusion, and stopping anticoagulation or reducing heparin, if necessary, should be given, as well as reversal of any underlying coagulopathies. Other intervening procedures, including operation, embolization, should be considerate when conservative treatment fail. Embolization may be necessary for pseudoaneurysm and active bleeding, because they usually do not spontaneously resolve and pseudoaneurysm have a tendency to rupture causing death.^[24]^ In the case reported by Hasegawa et.al,^[17]^ although angiography revealed active bleeding, embolization was not performed because the bleeding point was located in the proximal right hepatic artery, and as the patient showed no antegrade portal venous flow during TIPSS, embolization may result in infarction of the right lobe and hepatic failure occurring. But after conservative treatment, the hematoma gradually resolved and no evidence of pseudoaneurysm formation was found.

## 4. Conclusion

Intrahepatic hematoma due to TIPSS procedure is rare but potential life-threatening complication. The diagnosis should be considered in any patients with sudden right upper abdominal pain and hemodynamic compromise. Ultrasound and enhanced CT or MRI is the diagnostic modality of choice, which is rapidly attainable and can provide detailed information regarding the injured branch of hepatic artery. If suspecting hepatic artery injuries, including pseudoaneurysm and active bleeding, selective angiography is necessary. Early resuscitation and reversal of coagulopathy is essential, and embolization should be done in patients with pseudoaneurysm or unstable hemodynamic status. Whether it is necessary to embolization or stopping anticoagulation when sign of hepatic arterial injuries is found during TIPSS procedure is need to be further investigated.

## Author contributions

**Conceptualization:** Ziyao Cheng, Mingming Deng, Hailong Zhang.

**Data curation:** Ziyao Cheng, Jiayu Ju, Qingliang Zhu, Mingming Deng.

**Formal analysis:** Ziyao Cheng, Jiayu Ju, Qingliang Zhu.

**Supervision:** Hailong Zhang.

**Writing—original draft:** Ziyao Cheng, Hailong Zhang.

**Writing—review and editing:** Ziyao Cheng, Qingliang Zhu, Mingming Deng, Hailong Zhang.
